# Neurodevelopmental outcomes following intravitreal bevacizumab injection in Japanese preterm infants with type 1 retinopathy of prematurity

**DOI:** 10.1371/journal.pone.0230678

**Published:** 2020-03-20

**Authors:** Mitsuru Arima, Masato Akiyama, Kohta Fujiwara, Yujiro Mori, Hirosuke Inoue, Eiko Seki, Takahito Nakama, Shoko Tsukamoto, Masayuki Ochiai, Shouichi Ohga, Koh-Hei Sonoda

**Affiliations:** 1 Department of Ophthalmology, Graduate School of Medical Sciences, Kyushu University, Fukuoka, Japan; 2 Department of Pediatrics, Graduate School of Medical Sciences, Kyushu University, Fukuoka, Japan; Massachusetts Eye & Ear Infirmary, Harvard Medical School, UNITED STATES

## Abstract

**Purpose:**

The purpose of this study was to evaluate neurodevelopmental outcomes in 18-month old (corrected age) preterm infants who received an intravitreal bevacizumab (IVB) injection for the treatment of type 1 retinopathy of prematurity (ROP).

**Methods:**

In this ten-year retrospective study, we reviewed the medical records of patients who underwent ROP screening at Kyushu University Hospital. Among the patients who received IVB or laser photocoagulation (LPC) for the treatment of type 1 ROP, we included infants whose neurodevelopmental examination (the Kyoto Scale of Psychological Development [KSPD]) results at 18 months corrected age were available. Then, the effect of IVB on the developmental quotient (DQ) in each KSPD domain (Postural-Movement, Cognitive-Adaptive, or Language-Social domain) or the overall DQ was investigated by performing linear regression analysis.

**Results:**

Out of the 513 patients reviewed, 53 were included in the study. IVB and LPC were performed for 14 and 39 patients, respectively. Administration of IVB was significantly associated with neurodevelopmental delay in the Language-Social domain (p = 0.01). The observed association remained even after adjusting for gestational age and birth weight (p = 0.03).

**Conclusions:**

Administration of IVB may introduce a risk of developmental impairment of interpersonal relationships, socializations, and/or verbal abilities of preterm children. We recommended that preterm infants who received IVB undergo a neurodevelopmental reassessment during their school years or in adulthood.

## Introduction

Retinopathy of prematurity (ROP) is a retinal vasoproliferative disease that can lead to childhood blindness [1]. Younger gestational age (GA) and low birth weight (BW) are common risk factors for ROP progression [2], and the mean values of both are gradually decreasing worldwide [3]. Due to improvements in neonatal care [4], the overall number of ROP cases is currently decreasing, but there is a risk that the relative proportion of severe ROP cases that require treatment may increase in the future [5].

The initial standard treatment for ROP is laser photocoagulation (LPC) or intravitreal injection of an anti-vascular endothelial growth factor (VEGF) agent [6,7]. The therapeutic efficacy of LPC was established by the Early Treatment for ROP (ETROP) Study [7]. Evidence of the effect of an intravitreal bevacizumab injection (IVB) was presented by the Bevacizumab Eliminates the Angiogenic Threat of ROP (BEAT-ROP) Study [8]. Compared to LPC, the IVB treatment resulted in a lower recurrence rate of zone I ROP. Another advantage of IVB is that it is a less invasive treatment method for preterm infants, because the time needed for the IVB procedure is less than that needed to perform LPC. The off-label use of IVB is thus relatively established as a primary treatment method for severe ROP or general unstable conditions in preterm infants [9,10].

However, there is some concern that IVB can induce neurodevelopmental disabilities in premature infants [11]. This motivated us to investigate the effects of IVB on the developmental quotient (DQ) of Japanese preterm infants with ROP. We conducted the present study to determine whether administration of IVB poses a risk of neurodevelopmental delay by comparing the DQs of Postural-Movement, Cognitive-Adaptive, and Language-Social domains between the IVB and LPC groups of preterm infants with ROP.

## Patients and methods

### Patients

This study was performed in accordance with the tenets of the Declaration of Helsinki. After approval was obtained from the Institutional Review Board of Kyushu University Hospital, we conducted a retrospective chart review of the infants who underwent ophthalmic examinations from Nov 2007 to May 2018. We presented information of this study on our institutional website and informed all patients of their right to opt out. All data was completely anonymized so that patients could not be identified. ROP screening was performed on all infants born at ≤32 weeks GA or with a BW ≤1500 g. We administered IVB or performed LPC on infants who developed type 1 ROP. We defined type 1 ROP according to the criteria of the International Classification of Retinopathy of Prematurity Revisited: ROP at stage 2 or 3 in zone II with plus disease, ROP at stage 3 in zone I with or without plus disease, and ROP at stage 1 or 2 in zone I with plus disease [12,13].

After obtaining informed consent from the parents of each patient, we administered IVB (0.625 mg/0.025 ml) or performed LPC on the infants. We recommended IVB for patients with zone I ROP or a poor general condition. We used the Kyoto Scale of Psychological Development (KSPD) for the evaluation of the neurodevelopmental outcomes of our patients [14]. The KSPD is a face-to-face test that can assess the development of children from infancy in three domains: The Postural-Movement, Cognitive-Adaptive, and Language-Social domains. The overall DQ can be calculated using the DQs of these three domains [14]. As each of these individual DQs and the overall DQ are correlated with intelligence quotient (IQ) (the overall DQ has an especially strong correlation [r = 0.88]), the use of the KSPD also contributes to early interventions in pervasive developmental impairment [14–16]. Among the infants with ROP who underwent LPC or IVB, we selected the infants whose KSPD results at 18-months corrected age were available.

### Statistical analyses

All analyses were performed using JMP pro 13 (SAS, Cary, NC). We evaluated the relationship between IVB and the DQ of each of the three KSPD domains or the overall DQ by performing linear regression analysis. GA, BW, and DQ were treated as continuous variables; ROP zone, stage and IVB were treated as categorical variables. A two-sided p-value <0.05 was considered significant. To compensate for multiple comparisons, a Bonferroni-corrected p-value <0.05/4 = 0.0125 was considered statistically significant.

## Results

**Fig 1:**
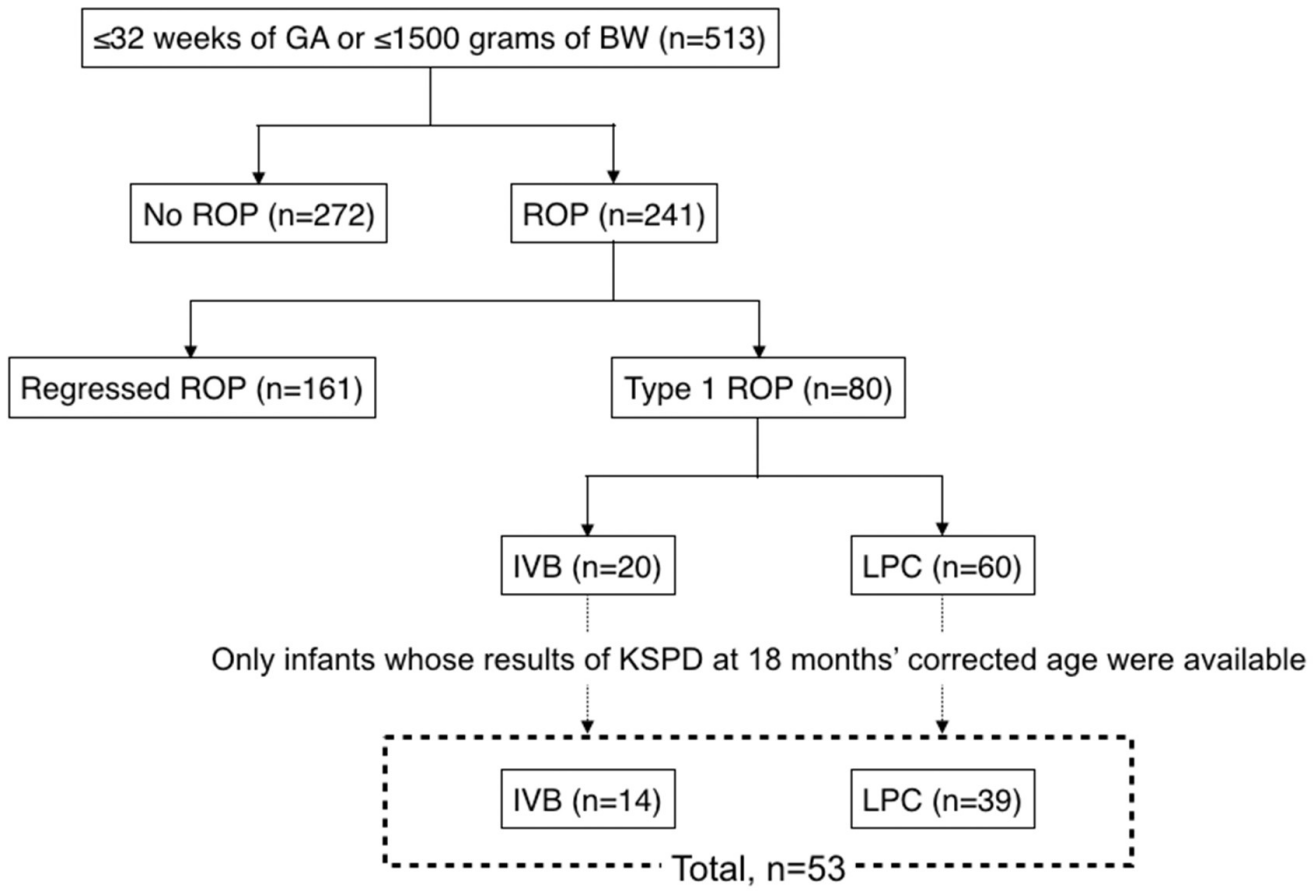
Patient flow diagram. Abbreviations: GA, gestational age; BW, birth weight; ROP, retinopathy of prematurity; IVB, intravitreal injection of bevacizumab; LPC, laser photocoagulation; KSPD, Kyoto Scale of Psychological Development test.

During the study period, 513 patients were examined, and 80 patients were found to have type 1 ROP (Fig 1). Among them, only 53 patients who we were able to obtain the KSPD results at 18 months’corrected age were analyzed in this study (Fig 1). The reasons for the exclusion of 27 patients are as follows: 1) Seven infants underwent a developmental examination other than KSPD; 2) Eight infants could not visit our hospital due to relocation; 3) Nine infants could not be examined because of severe central nervous system diseases such as epilepsy and cerebral palsy; 4) Two infants could not be examined because they were crying; and 5) One infant died before 18 months’ corrected age.

The characteristics of the 53 infants are summarized in Table 1. IVB was administered to 14 infants and LPC was performed for 39 infants. Compared to the infants in the LPC group, those in the IVB group had significantly lower GA and BW values (p = 0.006, p = 0.004, respectively). The percentage of patients with zone I ROP in the IVB group was significantly higher than that in the LPC group (p<0.0001, Table 1).

**Table 1:** Baseline parameters of the 53 infants enrolled in this study.

Baseline parameter	All (IVB + LPC)	IVB	LPC	p-value*
No. of patients	53	14	39	–
GA, weeks^†^	24.9 ± 1.7	23.9 ± 1.1	25.3 ± 1.7	0.006^‡^
BW, g^†^	633 ± 205	502 ± 154	680 ± 203	0.004^‡^
Zone, n (%)	Zone I: 16 (30) Zone II: 37 (70)	Zone I: 13 (93) Zone II: 1 (7)	Zone I: 3 (8) Zone II: 36 (92)	<0.0001^§^
Stage, n (%)	Stage 2: 12 (23) Stage 3: 41 (77)	Stage 2: 5 (36) Stage 3: 9 (64)	Stage 2: 7 (18) Stage 3: 32 (82)	0.26^§^

*Statistical analyses were performed to compare the IVB and LPC groups.

^†^Data are mean ± standard deviation,

^‡^—Student’s *t*-test,

^§^—Fisher’s exact test.

Abbreviations: IVB, intravitreal injection of bevacizumab; LPC, laser photocoagulation; GA, gestational age; BW, birth weight.

We investigated the relationship between IVB and the DQs of the three KSPD domains or the overall DQ. The results of linear regression analyses showed that IVB administration was significantly associated with the Language-Social domain DQ (Table 2; p = 0.0115); the association between IVB and the Language-Social domain DQ remained nominally significant even after GA- and BW-adjusted analyses (Table 3; p = 0.03).

**Table 2:** Comparison of the DQ in each domain and the overall DQ between the IVB and LPC groups.

Neurodevelopmental outcome	IVB	LPC	p-value*
Postural-Movement domain DQ	63.5 ± 21.5	75.1 ± 22.1	0.10
Cognitive-Adaptive domain DQ	66.9 ± 19.9	74.6 ± 16.8	0.17
Language-Social domain DQ	63.4 ± 19.2	77.7 ± 17.0	0.0115
Overall DQ	65.7 ± 18.6	74.7 ± 16.1	0.10

Data are mean ± standard deviation,

*Level of significance after Bonferroni correction: p<0.0125.

Abbreviations: IVB, intravitreal injection of bevacizumab; LPC, laser photocoagulation; DQ, developmental quotient.

**Table 3:** Multivariate analysis of the influence of GA, BW and IVB on Language-Social domain DQ.

Variable	β (mean ± standard error)	p-value
GA	-2.0 ± 1.8	0.27
BW, per 100 g	2.1 ± 1.5	0.16
IVB	-13.4 ± 6.1	0.03

Abbreviations: GA, gestational age; BW, birth weight; IVB, intravitreal injection of bevacizumab; DQ, developmental quotient.

## Discussion

This is the first study to investigate the influence of IVB on the neurodevelopmental outcomes of Japanese preterm infants with ROP. Our analyses revealed that treatment with IVB significantly decreased the Language-Social domain DQ at 18 months’ corrected age. Another study demonstrated that the Language-Social domain DQ was correlated with IQ (r = 0.81) [14]. Because this study is an analysis of small population in a single institution, further studies should be required to confirm the effect of IVB on the neurodevelopment in ROP infants. However, we believe that it is important to perform a long-term follow-up of the neurodevelopment of infants with ROP who were treated with IVB.

Morin et al. [11] reported that, compared to LPC, the use of IVB induces a risk of neurodevelopmental delay in ROP infants. The reason for this may be that IVB inhibits the development of the central nervous system (CNS) by suppressing the concentration of serum VEGF for over two months [17,18]. VEGF is an essential molecule in brain homeostasis as well as vasculogenesis [19,20]; thus, it is possible that prolonged VEGF suppression results in the inhibition of proper CNS development.

Although administration of IVB had an adverse effect on the patients in our study as well, Lien et al. [21] and Rodriguez et al. [22] indicated that there was no difference in the neurodevelopmental outcomes of their IVB- and LPC-treatment groups. The influence of IVB on CNS development thus remains controversial. The mean GA and BW values of our patients (23.9 weeks, 502 g) were low compared to those reported by Lien et al. (25.0 weeks, 749.6 g) and Rodriguez et al. (25.1 weeks, 698 g). The younger the GA and the lighter the BW, the more immature the cerebrovascular development will be [23]. Therefore, the specific characteristics of our patients may have been responsible for the difference in the neurodevelopmental outcomes recorded after IVB administration.

In our study population, IVB influenced the Language-Social domain DQ. There was a significant difference between the GA and BW values of the IVB and LPC groups. It is well known that these values influence neurodevelopment [23]. However, the association between IVB and the Language-Social domain DQ remained significant even after adjusting for GA and BW in the additional analyses. Although why IVB did not affect the DQ of the other domains is unclear, it is possible that long-term suppression of VEGF affects the neurodevelopment of premature infants by causing disruption of the brain microenvironment or inhibition of vasculogenesis.

Recently, the Ranibizumab versus laser therapy for the treatment of very low birthweight infants with ROP (RAINBOW) trial established the therapeutic efficacy of intravitreous injection of ranibizumab (IVR) [24]. It is also worth noting that unlike IVB, IVR did not reduce the concentration of serum VEGF [24]. If sustained VEGF suppression inhibits CNS development, administration of IVR may have a lower risk of inducing neurodevelopmental delay than IVB.

In conclusion, our analyses suggest that the administration of IVB may affect the development of interpersonal, social, and/or verbal skills in infants treated for type 1 ROP. Long-term observation of the development of infants with ROP who were treated with IVB is recommended.
